# An Overview of the Characteristics, Pathogenesis, Epidemiology, and Detection of Human Enterovirus in the Arabian Gulf Region

**DOI:** 10.3390/v16081187

**Published:** 2024-07-24

**Authors:** Mohammed Ayyub, Joshua George Thomas, Rawad Hodeify

**Affiliations:** Department of Biotechnology, School of Arts and Sciences, American University of Ras Al Khaimah, Ras Al Khaimah 72603, United Arab Emirates; mohammed.ayyub@aurak.ac.ae (M.A.); joshua.thomas@aurak.ac.ae (J.G.T.)

**Keywords:** enterovirus, echovirus, gulf countries, meningitis, pathogenesis, viral genome, detection, children

## Abstract

Enteroviruses are RNA viruses that initiate infections through the gastrointestinal (GI) tract and are associated with enteric illness in individuals of all ages. Most serious infections of enteroviruses are in infants and young children where it is the common cause of aseptic meningitis and other systemic diseases, leading to a high mortality rate. Enteroviruses belong to the small non-enveloped family of the Picornaviridae family. The virus can spread mainly through fecal–oral and respiratory routes. In the Arabian Gulf countries, the incidence of enteroviral infections is only restricted to a few reports, and thus, knowledge of the epidemiology, characteristics, and pathogenesis of the virus in the gulf countries remains scarce. In this minireview, we sought to provide an overview of the characteristics of enterovirus and its pathogenesis, in addition to gathering the reports of enterovirus infection prevalence in Gulf Cooperation Council (GCC) countries. We also present a summary of the common methods used in its detection.

## 1. Introduction

Enteroviruses are a large group of small positive-sense single-stranded RNA viruses belonging to the picornaviridae family [1]. Worldwide reports show a close association between enteroviruses and a wide variety of human disease. Enterovirus infections are presented with multiple diseases, ranging from mild illness such as croup, a sore throat, skin rash, fever, hand-foot-and-mouth disease (HFMD) to febrile illnesses, meningitis, myocarditis, neonatal hepatitis, and acute flaccid paralysis [2,3,4,5,6,7,8,9]. Although enteroviral infections can also affect adults, newborns and infants are at the highest risk for developing severe conditions, which can be associated with high morbidity and mortality within the first few weeks of life [10,11,12,13,14,15].

Enterovirus infections can spread mainly through the fecal–oral route as well as respiratory secretions from infected individuals [16]. The spread of the virus is common in schools, nurseries, and neonatal intensive units. The virus is also likely to spread by contact with an infected infant’s stool. As most enteroviral infections are asymptomatic or associated with mild illness, they can be overlooked, increasing the risk of developing more serious symptoms later [16].

In the Arabic Gulf region, the prevalence of enteroviral infections is only restricted to a few reports, and thus, public awareness about these types of infections remains minimal as well as the burden of the disease and quality control measures. Thus, combating enteroviral-initiated diseases requires a summary of reported cases, including geographical location, age, and associated disease. This is expected to be helpful to ensure sufficient resources are allocated to combat any possible future outbreaks. It is also helpful in bringing forward the need for further research on these kinds of viruses to be able to accelerate advances in designing new vaccines and antiviral treatments for affected individuals.

In this mini review, we (1) present an overview of the characteristics of enterovirus and its pathogenesis; (2) summarize relevant reports of enterovirus infection prevalence in the Gulf Cooperation Council (GCC) area; and (3) summarize common methods used in detection. 

## 2. Enterovirus Characteristics

Enteroviruses are positive-sense enveloped RNA viruses belonging to the picornaviridae family which is clustered under Baltimore class IV [17]. Enteroviruses comprise ten enterovirus species [18] including four that have been associated with infections in humans (A–D) [19]. Echoviruses, which belong to group B enteroviruses, are the largest subgroup of enteroviruses, comprising 63 serotypes [20]. Echoviruses 10 and 28 have been reclassified as reovirus-1 and rhinoviris-1, respectively. Echoviruses 22 and 23 have been reclassified as parechovirus type 1 and 2, respectively. In contrast, echovirus 34 was reclassified as variant coxsackievirus type 24 [21].

**Figure 1:**
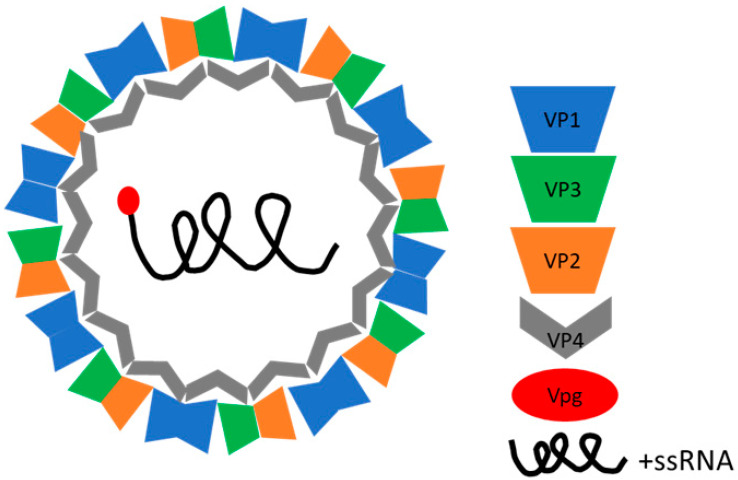
Structure of enterovirus. The cartoon shows the positioning of viral structural proteins VP1–4 and Vpg in an echovirus. VP1, VP2, and VP3 are located on the nucleocapsid surface as receptors for cellular recognition. VP4 is positioned within the inner surface of the capsid and facilitates RNA genome translocation, while Vpg acts as a primer for replication.

The genome of enteroviruses consists of about 7500 nucleotides packaged in relatively small particles of 24 to 30 nanometers. The icosahedral capsid is made up of 60 copies of four proteins: VP1, VP2, VP3, and an internal and comparatively unstructured VP4 (Figure 1) [22]. The former three are present on the surface of the nucleocapsid and serve as receptors for cellular recognition. VP4 is found inside the capsid and myristolyated at the N-terminus. It has been shown to play role in viral RNA genome translocation through myristoyl–VP4, forming hexameric membrane pores in the endosomal membrane to release echoviral RNA into the cytoplasm [23].

**Figure 2:**
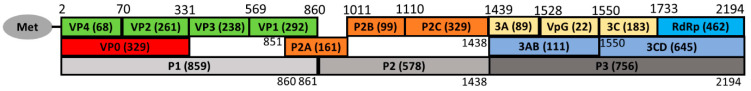
Schematic representations of the enterovirus genome and three polyprotein regions (P1, P2, P3). Viral structural proteins VP1–4. P2 and P3 regions contain all the nonstructural viral proteins. Viral proteases 2Apro and 3Cpro cleave the polyprotein into P1–P2 and P2–P3 regions, with 3Cpro further cleaving P1 to form capsid proteins and P2–P3 to yield various nonstructural proteins.

The RNA genome is attached to viral protein 3B (also known as Vpg) that acts as a primer for replication. The 5’ untranslated region contains an internal ribosomal entry site that helps in the translation of the viral genome. The viral genome is directly translated into a polyprotein containing three regions: P1 (859 amino acids), P2 (578 amino acids), and P3 (756 amino acids) (Figure 2). P1 regions encompass the viral structural proteins VP1–4, while P2 and P3 regions contain all the nonstructural viral proteins [24]. This polyprotein is then cleaved by viral proteases 2Apro and 3Cpro to separate polyprotein regions P1–P2 and P2–P3, respectively. Protease 3Cpro cleaves P1 to form the capsid proteins VP1 (292 amino acids), VP2 (261 amino acids), VP3 (238 amino acids), and VP4 (68 amino acids). In addition, it cleaves P2 and P3 to form nonstructural proteins 2A or protease 2Apro (161 amino acids), 2B (99 amino acids), 2C (329 amino acids), 3AB (111 amino acids), and 3CD (645 amino acids). 3AB is further cleaved by 3Cpro into 3A (89 amino acids) and VPg (22 amino acids), and 3CD is cleaved by the same protease to give protease 3Cpro or protein 3C (183 amino acids) and protein 3D or RNA-dependent RNA polymerase (RdRp) (462 amino acids) [24].

### 2.1. Infection, Entry, and Replication

**Figure 3:**
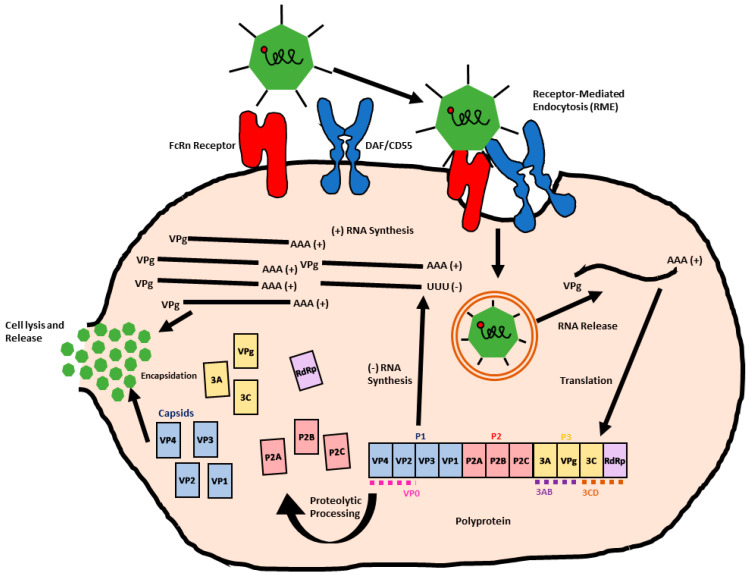
Enterovirus replication cycle. The cartoon illustrates the stages of echovirus infection by receptor−mediated endocytosis via FcRn and DAF/CD55 receptors, leading to RNA release, translation, proteolytic processing, genome replication and expression of structural proteins, viral packaging, and release.

Most enteroviruses including echoviruses initiate infection through the epithelia linings of the gastrointestinal tract, which allows the virus to disseminate to other places in the body [25]. Like other viruses, the replication cycle of enteroviruses is initiated by adhering and binding to the cellular receptor on host cells followed by receptor-mediated internalization. Subsequently, the viral genome is released and translated into a single polyprotein which is further processed to mediate the production of structural and nonstructural proteins. Finally, the newly replicated viral genome is packaged into new viral particles, which are released from cells through lysis or budding. For simplicity and due to multiple types of receptors used for the entry of different enteroviruses, we show the echovirus replication cycle as an example of the various steps of the enterovirus lifecycle (Figure 3). 

Enteroviruses use a wide array of receptors for cell attachment and entry [25]. 

For example, scavenger receptor B2 (SCARB2) and P-selectin glycoprotein ligand 1 (PSGL-1) were identified as host receptors that can bind enterovirus 71 and coxsackievirus A16 (CVA16) which belong to the species Enterovirus A (EV-A) [26,27]. Using a retroviral complementary DNA (cDNA) library from Jurkat T cells that are susceptible to EV71 infection, Nishimura et al. [26] identified PSGL-1 as a receptor that specifically binds EV71 virions. PSGL-1–EV71 interaction was blocked using KPL1, a monoclonal antibody, against PSGL-1 [28], confirming this protein as a functional receptor for EV71. The same study reported that CVA16 also uses PSGL-1 as a cellular receptor. Coxsackie B virus was reported to initiate infection by binding coxsackievirus and adenovirus receptor (CAR) after CAR cDNA transfection in nonpermissive hamster cells enabled coxsackie B virus attachment and infection [29]. On the other hand, the three poliovirus serotypes recognize CD155 (previously known as poliovirus receptor, PVR) [30,31].

Several different host receptors were identified to participate in echovirus entry, depending on the virus serotype (Figure 3). However, the receptors for most other serotypes are still unknown. It was demonstrated that integrin α2β1 acts as a receptor by echovirus serotypes 1 and 8 [32]. Another study has implicated a role for major histocompatibility complex (MHC) class 1 receptors in the entry of echovirus serotypes 12, 14, and 15 [33]. The human decay accelerating factor, DAF, (also known as CD55) was reported to be used by serotypes 7 and 12 [34], while serotypes 18 and 30 were reported to utilize the human neonatal fragment crystallizable (Fc) receptor (FcRn) [33]. This receptor is an evolutionary conserved receptor directing the transport of IgG to a location where it supports immunity [35,36]. A study by Zhao et al. [37], using CRISPR screening, demonstrated that FcRn is the functional uncoating receptor for echovirus 6. Furthermore, FcRn can be exploited by a large group of enterovirus B viruses for entry into many organs. The abundant expression of FcRn in the gut, placenta, and vascular endothelium suggests its important role in facilitating enterovirus B viruses in crossing both the blood–brain barrier and the blood–placenta barrier.

Previous studies predicted that echoviruses engage coxsackievirus and adenovirus receptor (CAR) for entry based on the close relation of these virus species [38,39,40]. However, a study by Vandesande et al. [41] in 2020 provided strong evidence that the Fc echovirus receptor (FcRn) is very crucial for successful echovirus 30 infection, while decay accelerating factor (DAF) receptors only act as coreceptors for attachment [41]. In this study, the authors utilized siRNA and pharmacological approaches to assess echovirus entry in a human lung cancer line, A549, and human rhabdomyosarcoma cells using immunofluorescence imaging of internalized labeled E30 virus. The study showed that the knocking down of the DAF receptor decreased but did not block E30 entry, while the knocking down of FcRn completely blocked echovirus entry, suggesting that FcRn is sufficient for a successful infection. The data from imaging were confirmed by the quantification of intracellular viral RNA using quantitative real-time PCR [41]. Interestingly, in this study, it was demonstrated that, unlike the entry of other enteroviruses, the entry of E30 is not dependent on clathrin-dependent endocytosis but rather occurs via raft-mediated endocytosis [40,42,43,44,45]. Once internalized to the endosomal system, virus uncoating leads to the release of viral-positive ssRNA into the host cell’s cytoplasm. The viral genome is missing the 5’ cap but contain 5′ and 3′ UTRs and is attached to viral protein 3B (Vpg), which acts as primer for replication [25]. The echoviral RNA has one open reading frame and can be translated using an internal IRES 5’ site to produce the polyprotein. The polyprotein is then cleaved by three viral proteases as described earlier to produce eleven proteins, VP1–4, 2A-C, and 3A-D. VP 1–4 are structural proteins and make up the viral capsid, while the other seven proteins 2A to 2C and 3A to 3D are essential for the replication process (Figure 2). Viral genome replication starts with the RNA dependent RNA polymerase (RdRp or protein 3D) on replication organelles that are built as a result of a rearrangement of cellular membranes. The RdRp induces uridylylation to VPg with the help of a cis-acting replication element, which is then anchored by the protein 3AB to the membrane [24]. The uridylylated Vpg is extended by RdRp to form a double-stranded RNA intermediate, which can be then used to synthesize positive-stranded RNA molecules which can undergo translation or replication or can be packaged into the viral capsids to form new viral particles. Protein 2B was shown to increase the permeability of ER in the host cell’s release of calcium into cytosol, which triggers the transport of viral ER-associated proteins to sites of viral genome replication. Protein 2C has been shown to contain similar motifs present in helicases and nucleoside-triphosphatase (NTPases) and have been shown to play role in viral RNA encapsidation [45,46,47].

### 2.2. Pathogenesis

Although the majority of enteroviral infections have mild or no symptoms, some may result in severe and sometimes life-threatening illness. Enteroviruses are associated with a wide array of diseases such as meningitis, paralysis, diarrhea, encephalitis, exanthema, myocarditis, pleurodynia, and various respiratory disorders [48]. In children and infants, enteroviruses, and specifically echovirus, are the most common cause of aseptic meningitis and encephalitis which is characterized by inflammation of the meninges of the brain and spinal cord or the brain itself. The initial mode of infection is mainly the gastrointestinal tract or respiratory system as the virus is spread through either aerosols or the ingestion of infected fecal particles. The replicated virus travels to the central nervous system, where it causes meningitis or other neurological problems due to neuronal injury. Because of this, the body increases the production of neutrophils, CD8 T cells, and monocytes. In the case of aseptic meningitis, echovirus increases the amount of TRIO (triple functional domain) protein levels in the neurons [49]. This protein contains a guanine-containing region that can be activated by echovirus, leading to the initiation of RhoA-targeted signaling. RhoA then causes the production of nitrous oxide which directly contributes to the death of the neural cells [49]. In addition to this, nitrous oxide affects the growth of the neural cells, ultimately leading to premature neuron formation. Another serious illness caused by enteroviral infection is acute flaccid myelitis (AFM), which is associated with paralysis and limb weakness that follow respiratory symptoms and fever. AFM cases have been associated with several different viruses including enteroviruses and echoviruses [50]. Furthermore, hand-foot-and-mouth disease (HFMD) can be caused by several types of enteroviruses. HFMD is commonly associated with sores or ulcers in the mouth, hands, and feet, with low-grade fever [51,52], and although most cases of HFMD resolve within a week, it can be associated with serious complications such as viral myocarditis, pulmonary edema, and aseptic meningoencephalitis [53]. In severe cases, enterovirus-associated HFMD can lead to death [54].

## 3. Incidence in GCC Countries

Several reports have been published about enteroviral disease in children and adults in GCC countries. However, to date, there has been no published review to summarize these reports. Here, we provide a concise summary of the incidence of enteroviral infections in GCC countries. Our aim is to inform policymakers, healthcare providers, and other stakeholders in Arab GCC states with the potential risk of enteroviral disease.

### 3.1. Qatar

In a study conducted from 2015 to 2018 on samples from patients with viral meningitis-like symptoms, there was a total of 503 cases of enterovirus (EV) disease caused by enteroviruses including echovirus. Among the reported cases, 428 cases affecting children from 0 to 9 years old, 45 cases in the case of adolescents from 10 to 19 years, and 29 reported cases of echovirus in adults from 19 to 60 years [55]. The clinical manifestations of EV infections included meningitis-like symptoms, fever and cough-like symptoms, and septic shock.

In another study by Ben Abid et al. in 2018 [56], 210 cases of viral CNS infection were reported, among which were 93 cases of enterovirus infections. In total, 47% of the reported CNS infections occurred in children younger than 16 years. Over half (51%) of the meningitis cases reported in this study were associated with enterovirus infections. 

An important study from Yassin’s lab on about 44,000 patients showing signs of influenza and other respiratory infections in Qatar between 2012 and 2017 reported 348 enteroviruses from various age groups. The clinical manifestations are like other respiratory pathogens, such as influenza virus and rhinovirus [57]. Similarly, six enteroviral cases were found from the years 2010 to 2011 [58]. Lastly, it was seen recently that the enteroviral strain EV-71 was able to cause neuromyelitis optica spectrum disorder in a 41-year-old male [59].

### 3.2. Bahrain

The Kingdom of Bahrain reported an echovirus epidemic that lasted from July 1995 to January 1996, where aseptic meningitis was frequently reported among subjects. The echoviral serotype associated with this outbreak was echovirus 30. The most affected group was children from 0 to 9 years, with 212 cases being reported. Moreover, 69 cases were reported in the age range from 10 to 19 years and 5 cases in adults aged from 19 to 60. A second study conducted in 1996 showed that 125 cases were found in children from 0 to 9 years old, 42 cases were reported in adolescents from 10 to 19 years, and 2 cases in adults from 19 to 60 years [60]. In 2002, a study reported that 10 cases were in toddlers from 0 to 4 years, 19 cases in children from 5 to 14 years, and 3 cases in children above 15 [61]. Samples from stool, sera, throat swab, and CSF confirmed infections by echovirus types 11, 6, 25, and 16, and nonpolio enterovirus.

### 3.3. Saudi Arabia

The Kingdom of Saudi Arabia reported 59 enteroviral cases in 1987. The strains included poliovirus 1, 2, and 3, echovirus 24, coxsackie b2, and coxsackie b3 [62]. The same group again reported 2 unknown enteroviral cases in 1989, 4 enteroviral cases (2 were unknown and 2 were poliovirus 2), 1 case of echovirus 6 infection, 1 case of coxsackievirus b1, and 2 cases of coxsackie virus B3 in 1991, 12 cases of echovirus 15, 25 coxsackieviruses A16, and B1, and polioviruses 2 and 3 in 1992, 11 cases of echovirus 9, 11 coxsackievirus A16, B1, and B4 in 1993, 24 cases of echoviruses 2, 4, 6, 17, 19, and 30, coxsackieviruses B2, B3, B4, and B5, and poliovirus 1 in 1994, and 29 cases of echoviruses 6, 11, and 24 and coxsackievirus B4, and polioviruses 2 and 3 in 1995 for a total number of 88 cases from 1991 to 1995 [63]. The clinical syndromes included upper respiratory infections, pneumonia, pharyngitis, and aseptic meningitis. From 2013 to 2014, three enteroviral respiratory cases were detected in the country [64]. In 2018–2019, a study conducted on pediatric patients (10 days old to 15 years) reported that 178 cases were associated with enterovirus infections and had respiratory symptoms [65]. Between 2018 and 2020, a study on 97 patients with aseptic viral meningitis patients showed 25% was due to enteroviral infections. Moreover, 66% of cases pertained to patients less than 4 years old and 18.6% of cases were reported in children between 5 and 17 years [66].

### 3.4. Kuwait

In Kuwait, a study over a three-year period on 147 infants and young children presenting symptoms of severe encephalitis, mild encephalitis, and febrile seizures confirmed enteroviral RNA in 37 (25%) of the cases. Echoviral infection was detected in 29 out of 37 cases. The major serotype was E9, followed by E30 and E9 [67]. In another study on 387 suspected aseptic meningitis patients, 49 cases of echoviral infection were found in infants from 0 to 2 years, 9 cases from children aged 2–4, and 2 cases from 4 to 12 years old. Similarly, the main echovirus types associated with the aseptic meningitis cases were serotypes E9, E11, and E30, coxsackie B4 and B5, and enterovirus 71 [68]. The identification of various serotypes was conducted by the DNA sequencing of VP4-VP2 regions. These regions from enteroviruses in Kuwait exhibited 92% to 97% homology with prototype strains of various specific enteroviruses. Another study showed that 34 neonates who displayed sepsis-like symptoms were affected by enterovirus [69].

### 3.5. United Arab Emirates

The United Arab Emirates reported 34 cases of viral meningitis due to enteroviral infections between 2000 and 2005 [70]. However, the distinct number of echoviral cases in this figure was not reported. In 2023, a study by Salim et al. [71] including 3098 children presented to medical facilities, served by a centralized microbiology laboratory between 1 January and 31 December 2019, reported rhinovirus/enterovirus (HRV) as the most prevalent infection. The screening was carried out for 17 viruses using FilmArray™ multiplex PCR (Biomerieux, Askim, Sweden). A study by Jeon et al. on 1362 patients who reported to a specialty hospital in the northern United Arab Emirates between 2015 and 2018 reported eight cases of enterovirus infections. Most of the cases were children between 4 and 7 years. The testing was conducted using a Multiplex rRT-PCR kit (Anyplex RV16, Seegene, Seoul, Republic of Korea) [72].

### 3.6. Oman

An unusual winter outbreak of enterovirus infections in Oman was reported between 2015 and 2016 [73]. This study reported 38 cases of non-polio enterovirus meningitis among Omani and non-Omani children less than 15 years, among whom 32% are less than one year old. Unfortunately, the study did not identify the enterovirus serotypes. The most common associated symptoms included headache, vomiting, neck pain, and fever. Other clinical manifestations included photophobia, irritability, and poor feeding. All patients exhibited a full recovery after a recovery period of 3–5 days after admission. The mild to moderate symptoms of the disease were attributed to the fact that none of the patients were less than 2 weeks old, suggesting more serious manifestations in neonates and infants [73].

## 4. Methods of Detection

The initial methods used to detect enteroviruses included propagation in cell cultures, followed by an observation of cytopathic effects (CPEs). Isolates were typed by a neutralization assay [74,75]. Hamagglutination is another assay that has been used due to the ability of several echoviruses to bind decay accelerating factor (DAF) found on erythrocytes as a cellular receptor [76]. Although the neutralization method is a common method for detection, it is time consuming and is challenged by its inaccuracy in detecting specific serotypes due to antigenic variation [77]. Thus, more specialized methods of diagnosis have been used. These methods include Neutralization assays, RT-PCR, and an indirect immunofluorescence assay. It is important to note that although these assays are commonly used for the detection or confirmation of specific enteroviruses, determining enterovirus genotypes requires the direct sequencing of specific PCR-amplified regions.

### 4.1. Neutralization Assay

Depending on the strain of the enterovirus, a variety of different antisera can be used for the detection of the virus through a neutralization assay. The earliest of this was the use of specific IgM antibodies for the detection of different echovirus strains which were EV6, EV21, EV30, and mainly EV33 [78].

In recent years, it has been seen that neutralization antibodies (nAb) of intravenous immunoglobulin (IVIG) are much better in the detection and neutralization of echovirus. However, they have been shown to have some issue with specificity as they may lead to cross-neutralization with different enteroviruses, which could cause misdiagnosis [79].

Another issue with this method is that commonly used pooled antisera Echopool 95 (EP95), which contains antibodies against echovirus 3–7, 9, 11, 14, 16, 17, 18, 22 (parechovirus 1), 24, 25, and 30 [80], cannot be used effectively for many other different strains of the enterovirus and it is often recommended to carefully interpret the viral breakthrough and go ahead with using specific antisera on the strain [81]. 

### 4.2. RT-PCR

This is a sensitive, accurate, and time-saving method for detecting echoviral strains. Echoviruses contain unique amino acid residues in the VP1 structural protein that differ among different serotypes. These unique VP1 sequences provide an excellent opportunity to make unique primers complementary to the VP1 sequence [82]. In addition, nucleotide sequences from genomic 5’UTR regions were used in the molecular detection of enteroviruses. 

Multiplex RT-PCR is highly recommended for unknown samples as it can detect two or more viruses in a single run of a PCR. Generally, enteroviruses encompass multiple viruses, such as echoviruses, coxsackievirus, and rhinovirus, which must be detected for an effective analysis. Multiplex RT-PCR allows for the specific and effective detection of all viruses while being cost effective. In the case of enteroviruses, specialized primers and probes are made that specifically attach to 5′non-translated regions (5′NTR) of the virus to avoid a cross-reaction with rhinovirus, which is also classified as an enterovirus [83].

In the Gulf region, most studies have been carried out using commercial RT-PCR kits, such as FilmArray™ multiplex PCR (Biomerieux, Askim, Gothenburg, Sweden) [71], Anyplex RV16 (Seegene, the Republic of Korea) [72], and the FTDResp21 kit (Fast Track Diagnostics, Sliema, Malta) [58]. The assay used for multiplex RT-PCR is highly dependent on the viruses that can be expected to be detected from a particular sample, and thus, it is solely dependent upon the researcher’s choice. Moreover, researchers can incorporate any other commercially available assay for detecting specific enteroviruses. 

It is noteworthy to add while RT-PCR is useful for the rapid detection of enterovirus analysis through its amplification of specific regions of 5′UTR and mostly VP1 [84], phylogenetic analysis and correct genotyping is determined by the sequencing of VP1 or VP2 [85].

### 4.3. Indirect Immunofluorescence Assay (IFA)

This requires initially virus isolation from the sample and propagation in an appropriate cell culture using cell lines such as RD (human rhabdomyosarcoma), HEp 2 (human larynx carcinoma epidermal), Vero (green monkey kidney) [86]), primary rhesus monkey kidney (RMK), human lung carcinoma (A-549), and diploid lung fibroblast (MRC-5) [87]. Once the virus propagates, it will destroy the target cells, producing a visible effect known as the cytopathic effect (CPE). It is recommended to use IFA when the cell culture achieves 75% CPE, where commercially available specific monoclonal antibodies are used to detect the enterovirus under a fluorescence microscope [87]. The major challenges in using IFA in enterovirus detection are its costs, time consumption, and the availability of specific antibodies.

## 5. Conclusions

The knowledge and awareness surrounding enterovirus infections are constantly developing, albeit at a pace not sufficient in comparison to the reported cases of infection. Although human enteroviruses are ubiquitous viruses, they have been associated with a wide array of diseases, ranging from mild symptoms to severe and life-threatening conditions, especially in infants and children. A greater concern is the increased prevalence of these diseases among infants and newborns and the potential serious symptoms that could be associated with echoviral infections. This, along with the probability of enterovirus infections being asymptomatic or mildly symptomatic, poses a challenge as these diseases may be unnoticed till more serious symptoms develop. Therefore, the early detection and intervention of echoviral infections in children are necessary. The prevalence of enterovirus infections in GCC countries highlights the importance of understanding regional epidemiology. Given the evidence that infections in humans have been mostly associated with enterovirus species A and B, it is important to keep in mind that any of human enterovirus species (A–D) can be highly pathogenic and have epidemic potential. Hence, further research is needed to better understand the molecular aspects of enterovirus infections in the Gulf region, increase the pace of the development of new vaccines and antiviral treatments, and improve the overall vigilance of GCC healthcare systems.
